# Nanoparticulated polymeric composites enfolding lithium carbonate as brain drug in persuading depression: an in vivo study

**DOI:** 10.1007/s40204-017-0076-8

**Published:** 2017-11-07

**Authors:** A. Anita Margret, V. Violet Dhayabaran, A. Ganesh Kumar

**Affiliations:** 1 Department of Biotechnology and Bioinformatics, Bishop Heber College, Tiruchirappalli, Tamil Nadu 620 017 India; 2 Laboratory of DNA Bar Coding and Marine Genomics, Department of Marine Science, Bharathidasan University, Tiruchirappalli, Tamil Nadu India

**Keywords:** Lithium carbonate, Depression, Chitosan, Nanocomposite, Drug delivery

## Abstract

Lithium carbonate is considered an effective drug against mania and acts as a mood stabilizer. It is found that it enhances antidepressants targeting depression, consequently it is prone to have risk factors that leads to adverse effects. The study is devised in confronting depression under nanoscale by preparing nanocomposites which is a matrix of biopolymer chitosan that encapsulates lithium carbonate by ionic gelation method. This facilitates the drug delivery in a regulated manner targeting the therapeutic action with a limited dosage that lessens the side effects in the course of treatment. The drug polymer interaction was validated by XRD studies, whereas the morphology and size characterization by SEM and zetasizer. The average particle size was determined as 193 ± 0.18 nm with a positive zeta potential of 37.9 mV. The in vitro drug release patterns of nanocomposites were comparatively assayed with the standard lithium carbonate which rendered a controlled release in its profile. The in vivo investigation by animal despair studies bestowed a significant difference in the duration of immobility during force swimming and tail suspension tests. These results were substantiated with histopathological examinations of cerebral cortex region which showed mild cellular edema, degenerative changes and lymphocytic infiltration when compared with the control groups. Consequently, the efficacy of nanocomposites encased with lithium carbonate fortifies targeted drug delivery and restrains adverse effects by endorsing it as a lead compound in brain drug developmental research.

## Introduction

Depression is considered as a threat that burdens the world intensively (GBD 2010). Individuals affected with this mental ailment are with disrupted mood that affects their basic subsistence of life (Murray et al. 2013). Recurrent mood disorders in depressive episodes can cause devastating effects which are associated with high risk of self-harm and suicide. These psychological conditions are remedied with antidepressant medications and psychotherapies. Lithium carbonate is an inorganic chemical salt that is therapeutically effective against mania and acts as mood stabilizer. It is listed as a significant and essential medication by the World Health Organization required for the vital health system (WHO 2013). It is also widely used for the treatment of psychiatric disorders (Sadock et al. 2009) and exerts neuroprotective effects (Fukumoto et al. 2001).

Conversely, the exact mode of action as a mood stabilizing agent is not established (Clough et al. 2014) but it is reported to interfere with transmembrane sodium exchange in nerve cells by affecting sodium, potassium-stimulated adenosine triphosphatase (Na+, K+-ATPase) stimulus. It has accounted to alter the release of neurotransmitters by affecting cyclic adenosine monophosphate (cAMP) concentrations which blocks inositol metabolism that results in depletion of cellular inositol and inhibition of phospholipase C-mediated signal transduction (Hardman et al. 2001). Extensive increase of protein kinase C (PKC) activity within the brain elevates the maniac condition and lithium carbonate augment sodium valproate to inhibit the excess accumulation of the enzyme (Yildiz et al. 2008). Learning and memory activities are enhanced by the brain-derived neurotrophic factors (BDNF) and lithium promotes neurogenesis and long-term potentiating (LTP) that can restore deficits (Leyhe et al. 2009).

Therapeutic drug monitoring is an essential criteria that distinguishes drugs in particular to their adverse effects and the curative effect of lithium carbonate is directly related to its concentration in serum, which ranges between 0.5 and 1.5 mEq/L (Christian 2002). The exceeded frontier of therapeutic level resulted in lithium toxication and is prone to have risk factors such as loss of consciousness; muscle tremor, epileptic seizures, and pulmonary complications (Smith et al. 2013). Delivery and distribution of drugs in a refined conduct can avoid adverse effects. The field of drug research has established nanoparticulate drug delivery systems using biodegradable and biocompatible polymers to overcome these challenges (Bala et al. 2004). Nanodrugs are advantageous for they have specific control, sustained and targeted release characteristics.

Patient compliance can be improved using formulations at nanoscale that can reduce the dosing frequency of drugs that can develop the probability of developing low cytotoxicity and reduced side effects. Chitosan is a prominent cationic polyelectrolyte biopolymer that has the potential of serving as an absorption enhancer across intestinal epithelial. The mucoadhesive and permeability-enhancing property of the polymer adhere to the mucosal surface and transiently opening the tight junction between epithelial cells (Qian et al. 2006). Drugs targeting central nervous system (CNS) are being challenged by the complicated mechanism of the blood–brain barrier (BBB) which is a system of layers of cells at the cerebral capillary endothelium connected by tight junctions (zonulae occludens) intended to protect brain (Greenwood 1992).

Therefore, there is a sturdy desire to develop a drug delivery system that permits the drug to bypass the BBB which is considered as a hurdle and results in the inability of some small and large therapeutic compounds to the targeted site. The polymeric technology nanocomposites unified with nanometric compound can consent drug delivery with low brain concentrations thus greatly enhancing the therapeutic effect on brain diseases (Dikpati et al. 2012). The study incorporates a nanoapproach in synthesizing biopolymeric nanocomposites encapsulating lithium carbonate by substantiating it with an animal despair and histopathological study to endorse the effect of nanocapsulated drug as an effective brain drug.

## Experimental

### Materials and methods

#### Drugs and reagents

Chitosan with the deacetylation degree (DD) of 95% and molecular weight (Mw) of 360 kDa was purchased from Panacea Biotech (Punjab, India). Lithium carbonate was bought from Sigma-Aldrich Corporation (St. Louis, USA) and the standard antidepressant drug was obtained from Cadila Pharmaceuticals, (Ahmedabad, India). All other chemicals and solvents were of analytical reagent grade.

### Preparation of nanocomposites by ionic gelation method

Chitosan was dissolved in 1% acetic acid solution to form a concentration of 10 mg/mL of solution, which was diluted with water to various concentrations: 1.0, 2.0, 3.0, 4.0, and 5.0 mg/mL. Subsequently, the TPP prepared at 0.25% w/v, was added dropwise to the chitosan solution and stirred consistently.

Drug (lithium carbonate)-loaded chitosan nanoparticles (CS/NPs) were prepared based on ionic gelation (Calvo et al. 1997) of chitosan with sodium tripolyphosphate anions (TPP). Chitosan was dissolved in 1% acetic acid solution to form a concentration of 10 mg/mL of solution, which was diluted with water to various concentrations: 1.0, 2.0, 3.0, 4.0, and 5.0 mg/mL with a mild and constant stirring (magnetic stirrer for 24 h under room temperature) until the suspension became clear and transparent. Subsequently, the prepared TPP solution at a concentration of 0.25% w/v was added dropwise to the chitosan solution (0.1% w/v) and stirred consistently. The drug-loaded CS/NPs were prepared by dissolving 10 mg of lithium carbonate in 5 mL of 2% w/v Tween 80 solutions, and added to the prepared chitosan solution. The suspension was incubated for 1 h at room temperature and centrifuged (Remi R-4C, India) at 14,000 rpm for 30 min at 10 °C. The settled NPs were re-suspended in distilled water for further formulation development and analysis. Further, a blank solution was also prepared without the drug and used as a control.

### Characterization of nanocomposite formulations

X-Ray diffraction patterns of lithium carbonate-loaded CS/TP nanoparticles were compared with that of standard samples of lithium carbonate, chitosan and the prepared blank. The powder X-ray diffraction pattern of drug was recorded by an X’Pert Pro X-ray diffractometer (PAN analytical BV, The Netherlands) operated at a voltage of 40 kV and a current of 30 mA with Cu Kα radiation in a *θ*–2*θ* configuration. Particle morphology was examined by a Hitachi scanning electron microscopy (S-4500) and, the samples were immobilized on copper grids. They were dried at room temperature, and examined without being stained. The particle size range of the nanoparticles along with its polydispersity was determined using a particle size analyzer (90 Plus Particle Size Analyzer, Brookhaven Instruments Corporation). Particle size was approached based on measuring the time-dependent fluctuation of scattering of laser light by the nanoparticles undergoing Brownian motion. The zeta potential of nanoparticles was measured on a zeta potential analyzer (Brookhaven, USA). For zeta potential measurements, samples were diluted with 0.1 mM KCl and measured in the automatic mode. All measurements were performed in triplicate.

### Drug loading and encapsulation efficiency

The drug loading and encapsulation efficiency was determined by separating the nanoparticles from the aqueous medium containing free lithium carbonate by centrifugation at 20,000 rpm at 4 °C for 60 min. The amount of free lithium carbonate in supernatant was quantified by measuring absorbance at 274 nm using a Shimadzu UV–vis (1601PC, Japan) spectrophotometer. Drug encapsulation and loading efficiency were then calculated using Eqs. (1) and (2), respectively. All measurements were performed in triplicate and observed in percentage (%).

1 $$ {\text{Drug encapsulation}}\, (\% ) = \frac{{({\text{Initial concentration of lithium carbonate }} - {\text{concentration of lithium carbonate in supernatant}})}}{\text{Initial concentration of lithium carbonate    }} \times 100 $$

2 $$ {\text{Loading Capacity}}\, (\% ) = \frac{{({\text{Total  lithium carbonate }}{-}{\text{free lithium carbonate}}) \times 100}}{\text{Nano particle weight}} \times 100 $$

### In vitro drug release study

In vitro release of lithium carbonate from CS/TPP nanoparticles was studied (Hu et al. 2002) by re-dispersing separated nanoparticles (10 mg) in 2.5 mL freshly prepared phosphate buffer of 7.4 pH, in a dialysis membrane bag with molecular weight cut off at 5 kDa. The dialysis bag was placed in 50 mL of phosphate buffer of pH 7.4. The entire system was kept under magnetic stirring. The release medium of 4 mL was removed and replaced by fresh buffer solution at regular time intervals. The amount of drug in the released medium was evaluated from the absorbance measured at 274 nm. All the release studies were conducted in triplicate with a standard lithium carbonate and mean values were taken.

### Grouping of the animals

The animals were accommodated under standard experimental conditions according to the rules and regulations of institutional ethical Committee for the Purpose of Control and Suspension of Experiments on Animals (CPCSEA) under Ministry of Animal Welfare Division, Government of India, New Delhi (ref no. BDU/IAEC/2014/NE/39). Female Albino mice with an average weight of 22–25 g (16 mice) were experimented (temperature of 24 ± 3 °C, humidity 40–60% with 12 h light and dark cycles) with a free access to food and water ad libitum. The animals with uniformity in their weight were acclimatized for a period of 7 days prior to study. They were randomly divided into four groups assigned as positive control (depressive without treatment), negative control (normal) and experimental groups (standard drug-lithium carbonate) and experimental group (nanocomposites). The animals in positive control grouped as 1 were administered with saline as vehicle (1 mL of 0.9%).

### Induction of depression and treatment schedule

The animals were induced to a depressed state by injecting methyl isobutyl ketone (100 mg/kg weight, i.p.) regularly for 2 weeks. Further, the induction was also carried out (2 weeks) with the animal despair studies during the final week and the test sessions (1 week) were performed during the following week (1st, 3rd, 5th and 7th day) to access their behavioral patterns. The treatment phase (dosage as depicted in Table 1) is pursued for another 1 week immediately after the test session and the final despair studies were conducted on the last day (14th day) after which the animals were sacrificed for histopathological studies.

**Table 1:** Animal protocol design represents groups of animals and their test drug dosages administered to mice

Groups	Treatment	Dose	Dose volume	Routes of administration
I	Disease control (depression induced)	Saline	1 ml	Oral
II	Normal	**–**	**–**	**–**
III	Standard lithium carbonate	100 mg/kg 300 mg/kg	1 ml	Oral
IV	Test nanocomposite	10 mg/kg	1 ml	Oral

### Modeling animals for depression studies-animal despair assay

#### Forced swim test (FST)

To assess the antidepressant activity of the nanocomposite, forced swim test (Porsolt et al. 1978) was conducted in three trials. The first trial was experimented with the depressed mice which have not undergone the treatment, the second on non-depressed normal mice and the third on the treated mice with the nanocomposite and standard drug. Each animal was placed individually in a 5 L glass beakers, filled with water up to a height of 15 cm and were observed for a duration of 6 min. During the test session, the immobility time, swimming and climbing periods were observed. The duration of immobility was recorded during the last 4 min of the observation period. The mice will try to escape from this stress induced and will try to climb. It would remain immobile and renounce its attempts. This is motionless floating of the animal with its head faced above the water level is calculated as the period of depressive-like state. The water was changed periodically after each session of experiments.

#### Tail suspension test (TST)

Tail suspension test is another animal despair study adopted (Cryan et al. 2005) to access the antidepressant activity. The animals were hung by their tails on a plastic string 75 cm above the surface with the help of an adhesive tape. The animals tend to climb up and attempt to bounce of the sudden stress after a series of efforts it will tend to be immobile, which is considered as a depressive behavior. This duration of immobility is observed in the animal despair study for 6 min. Mice were considered to be immobile only when being hung passively and were completely motionless.

### Histopathological studies

Subsequent to the animal despair studies (within 2 h), the mice were anesthetized, whose brain was dissected and sectioned into two halves. One half of the brain was kept in 50 mL-tube filled with 10% formalin. The brain tissues were dehydrated with alcohol through and clearing them in xylene, which are finally embedded in paraffin wax (mp 58–60 °C). Transverse sections of 5-μm thickness were cut on a rotary microtome. These sections were stained with Ehrlich’s hematoxylin and eosin in alcohol, dehydrated in alcohol, cleared in xylene, and examined microscopically to execute the histopathological studies.

## Results and discussion

### Formulation and synthesis of nanoparticles

The chitosan–TPP nanoparticles as blank was initially synthesized, based on which the test sample lithium carbonate was loaded. The nanoparticle synthesis was carried out at ambient temperature where, the preparation was simple, rapid, and reliable. CS/TPP nanoparticles were obtained spontaneously under very mild conditions. The formation of nanoparticles is only possible within some moderate concentrations of chitosan and TPP. The gelation between TPP solution of 1 mg/mL and chitosan solution of 1–3 mg/mL resulted in an opalescent suspension which further was examined as nanoparticles. This served as a blank which can be contrasted against the test component. Since lithium carbonate was hydrophilic in nature, there was a formation of uniform suspension. Nanoparticles were prepared by electrostatic interaction, when oppositely charged macromolecules were mixed together. The triumph of this concept relied on intermolecular linkages created between the negatively charged groups of TPP with that of positively charged amino groups of CS. This principle augments the encapsulation of lithium carbonate along with the polymer. Nanocomposites were synthesized in five different concentrations (1.0, 2.0, 3.0, 4.0 and 5.0 mg/mL) of chitosan where the third concentration (3 mg/mL) was optimized as standard. This was authenticated by the visual observation of opalescent suspension when the chitosan and the TPP concentrations were appropriate at the third concentration.

### Physicochemical characterization of prepared nanocomposite

**Fig. 1:**
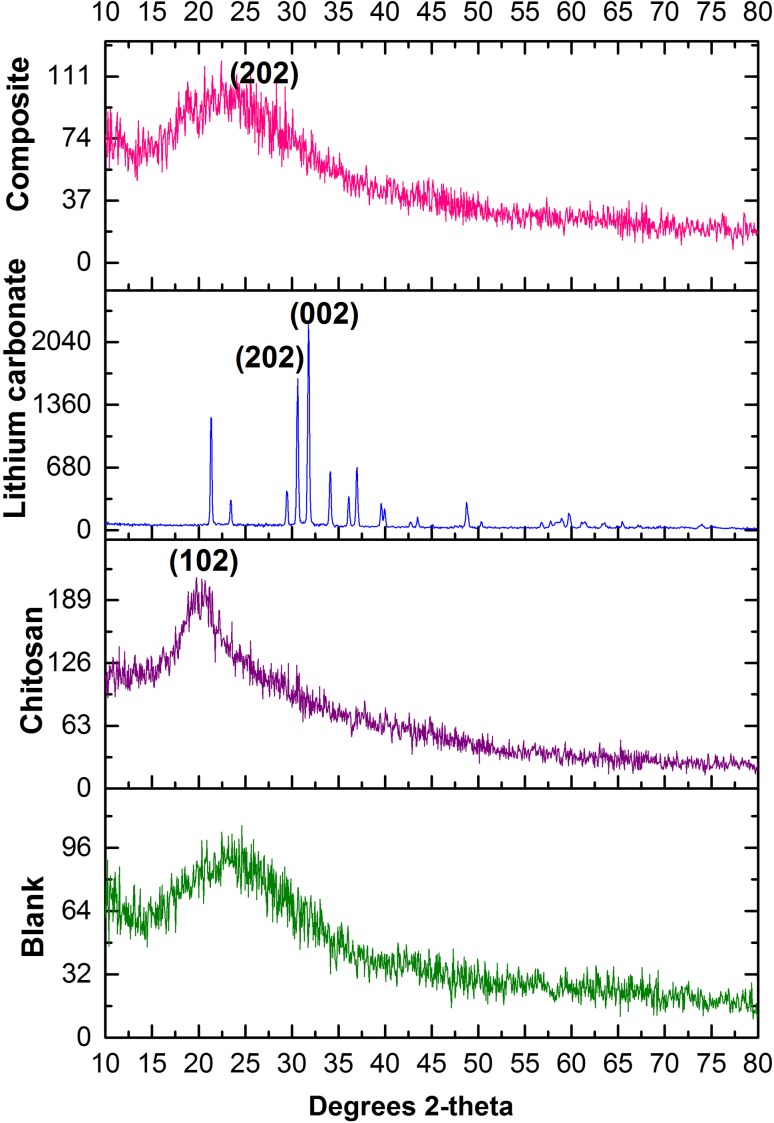
X-ray diffraction patterns of the standards (**a** chitosan; **b** lithium carbonate) encased as nanocomposites (**c**) along with blank (**d**)

Physicochemical characterization studies were made to analyze the drug–polymer interaction and the morphological features. The XRD pattern depicts the interaction of drug with the polymer which is crystalline in nature. The lithium carbonate-loaded chitosan/TPP formulation exhibited a transformation in its texture to amorphous elucidating crystallinity due to the incorporation of lithium carbonate with the polymer. The crystallographic structure and grain size are identified by comparing diffraction data against a database of known materials. Figure 1a–d illustrates the XRD patterns of chitosan, lithium carbonate, nanocomposite and blank (formulation devoid of lithium carbonate). The peaks obtained from Fig. 1b had sharp peaks where 30.5° and 31.7° corresponded to JCPDS no. 87-0728, with contest index of 200 and 002 planes in standard lithium carbonate. The peak obtained in nanocomposite sample had an index of 202 at 30.5° which attested the integration of the polymer with standard lithium carbonate (Fig. 1c).

**Fig. 2:**
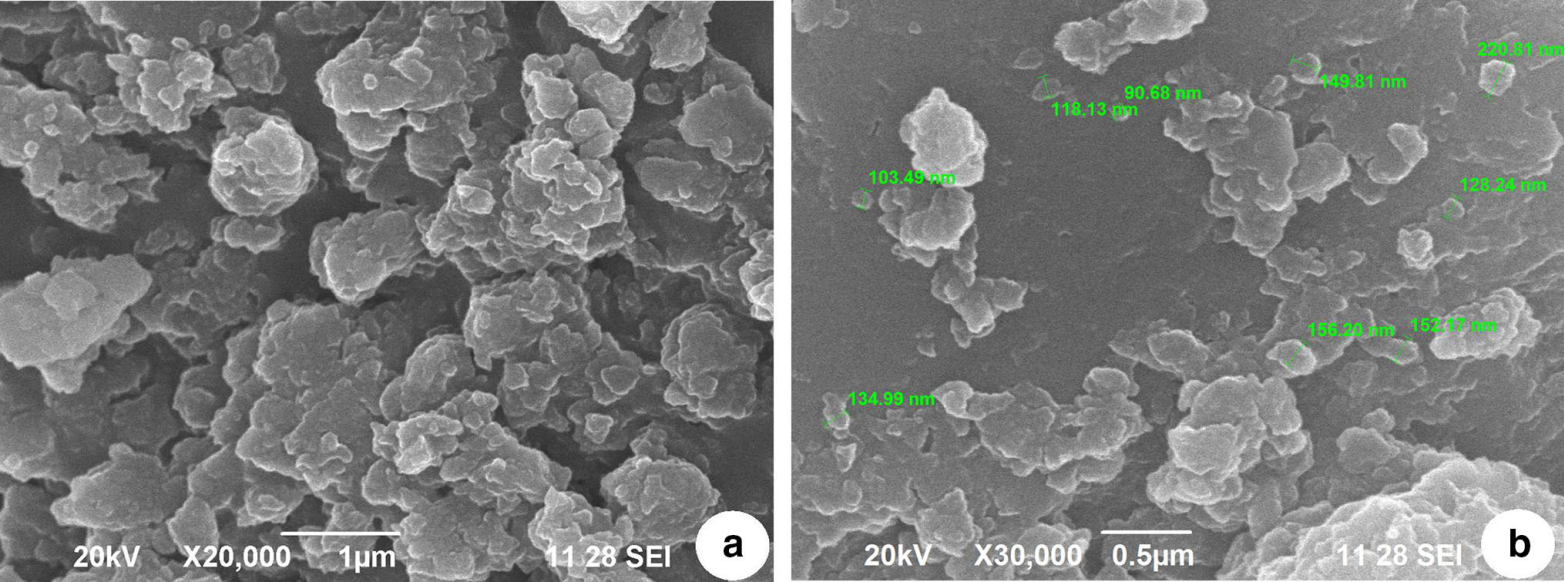
SEM micrographs of nanocomposites at different resolutions

The peak widths and shapes describe the deviations from a perfect crystal that are more amorphous. The width was employed to calculate its average crystallite size using Scherrer equation which was found with an average size of ∼ 143.27 nm. Chemical structure of compound was found through lattice parameter using X-ray diffractometry and the chitosan is depicted with the strongest peak obtained is at 20.1° (Fig. 1a) which is the typical identification for the semi-crystalline structure of a biopolymer (Islam et al. 2011; Zhang et al. 2012). The blank suspension (Fig. 1d) that consists of CS/TPP nanoparticles were in the form of amorphous and did not show the intensity of characteristic peaks of chitosan (Wan et al. 2003). Shape and surface morphology of prepared nanoparticles were evaluated by scanning electron microscope. Figure 2a, b shows the morphological characteristics of nanoparticles by scanning electron micrograph at different resolutions.

**Fig. 3:**
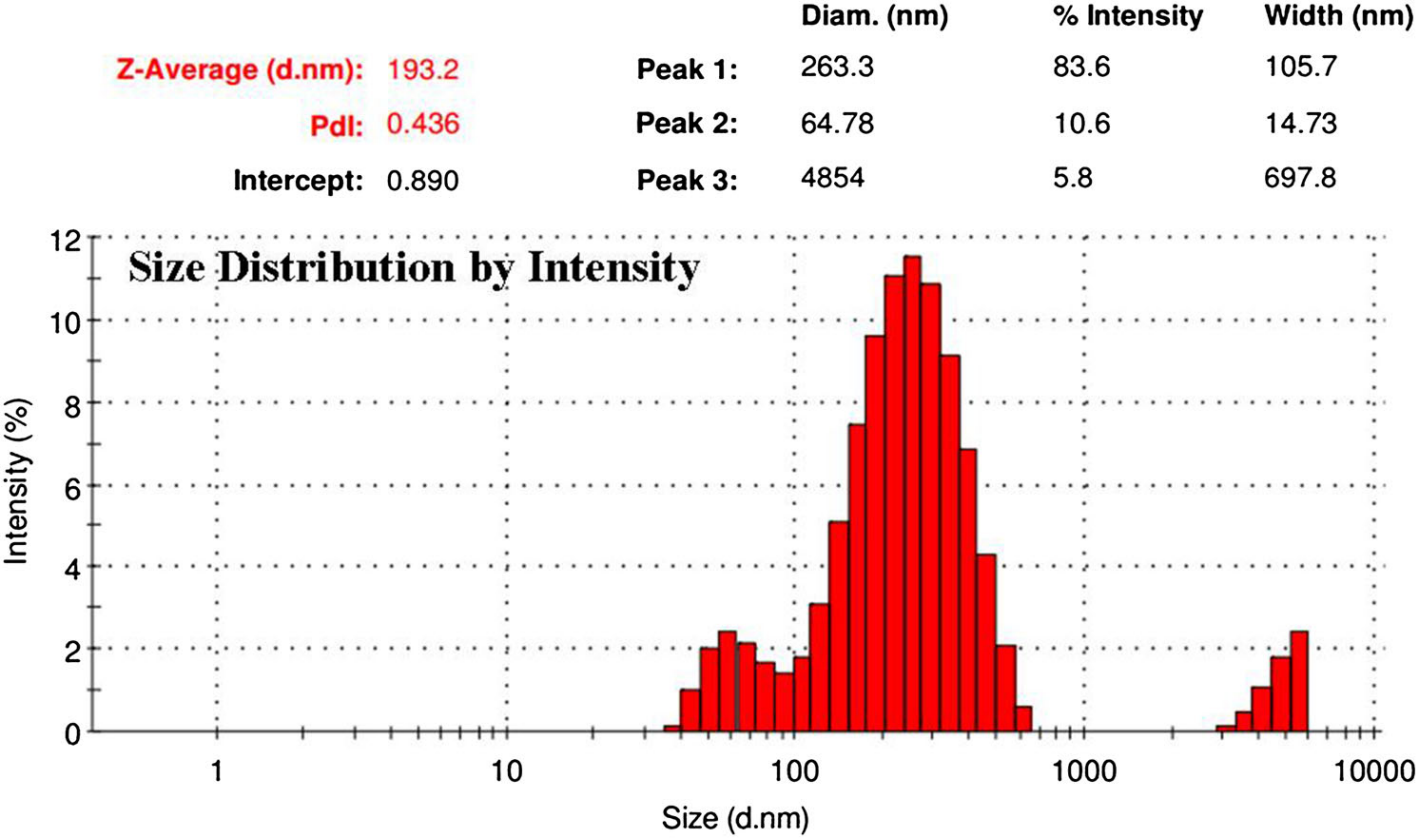
DLS measurement to determine the size distribution of nanocomposites

**Fig. 4:**
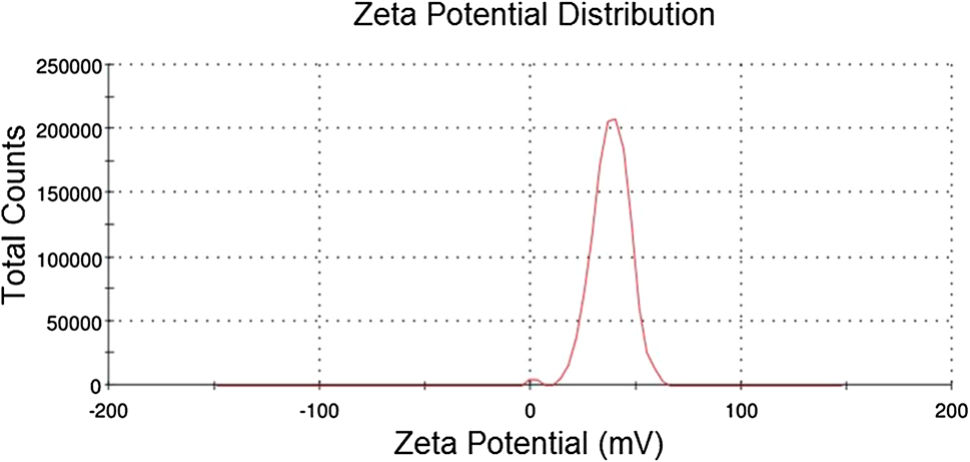
Zeta potential analysis to determine the stability of nanoparticles

The shape and surface morphology of the nanocomposites was evaluated and the microscopic images showed irregular mass with various size compositions (90.68–220.81 nm) owing to the non-homogenous, non-smooth surface with straps and shrinkage of the polymer chitosan (El-Hefian et al. 2010). The dynamic light scattering (Fig. 3) sustains the nanocomposite particle size of SEM analysis that revealed scattering intensity corresponding to an average diameter of 193.2 nm. The zeta potential value of 37.9 Mv (Fig. 4) reveals information regarding the surface charge and good colloidal stability of the prepared nanoparticles. The positive zeta potential is due to the formation of residual amino groups by the interaction of chitosan with charged (positive) lithium, which reflect only a part of the amino groups that are neutralized thereby influencing brain drug targeting (Beduneau et al. 2007). The entrapment efficiency was estimated to be 87 ± 1.21% which denotes the encapsulation of standard lithium carbonate was encapsulated in the nanoparticles. Drug loading efficiency was calculated to be 28.87% which inferred the content of lithium carbonate by weight (1 mg of nanoparticle consisted of 0.28 mg). Loading capacity of the nanoparticles was affected by the initial lithium carbonate concentration in the CS solution and the amount of lithium carbonate incorporated. The mechanism of lithium carbonate association to chitosan nanoparticles was mediated by an ionic interaction between both chitosan and lithium carbonate. The electrostatic interactions between the carboxyl groups of the drug and the amino groups of chitosan play a role in association of lithium carbonate to the chitosan nanoparticles.

**Fig. 5:**
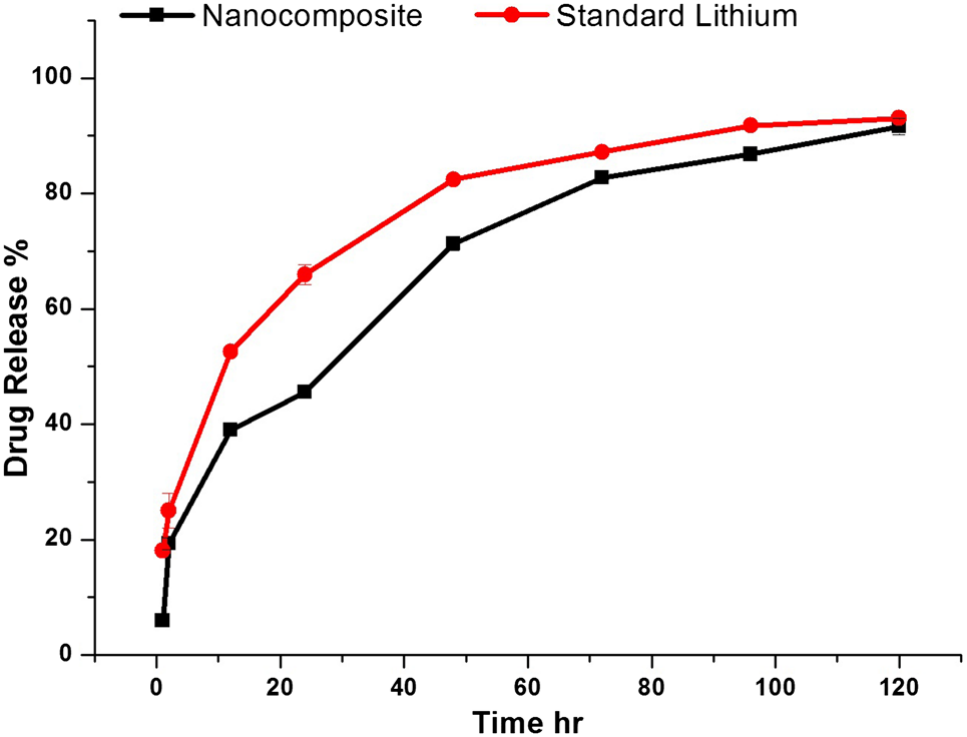
Relative release profiles of the nanocomposite system with standard lithium carbonate

The release rates of lithium carbonate incorporated in the polymeric matrix and standard lithium carbonate solutions at various time intervals were calculated and presented in Fig. 5. The release process consisted of three distinct features such as penetration (determines the dosage), dissolution and diffusion. The release profile of lithium carbonate from the chitosan nanoparticles decreased with time by a slow and sustainable release when compared to the standard lithium carbonate. In general, the drug release is due to the dispersal of drug molecules through the matrix or due to deprivation of polymeric matrix (Mu and Feng 2003). The initial burst release of drug molecules from nanoparticles is endorsed to the presence of drug molecules near the edge of the nanoparticles, which diffuse in the surrounding medium due to rapid penetration of release medium into the nanoparticles. The release rate was observed to be slow for lithium carbonate-encased CS/TPP nanoparticles as compared with the standard lithium carbonate. This is due to the presence of surface crosslinking of the polymeric matrix which produces intrusion to the diffusion of drug molecules in release medium. However, the burst release is mainly due to the surface-absorbed drugs.

A polymeric drug carrier primarily diffuses the drug molecules and chitosan as a mucoadhesive, cationic polymer assists the drug to diffuse easily during the initial incubation period (Zhou et al. 2001; Berscht et al. 1994). The constituents of nanoparticle acted as control release when compared to the standard sample. A spontaneous elevation in the drug release profile was observed at 70 h and was relatively close to the standard with a difference of 5%. The amount of drug released from the dialysis bag attained its maximum proficiency after 120 h which was similar to the standard lithium carbonate though there was a rapid increase in the initial burst release. The smaller size of the drug was a significant parameter which facilitated its transfer and the encased polymer provided control release mechanism.

### Assessment of depression

**Fig. 6:**
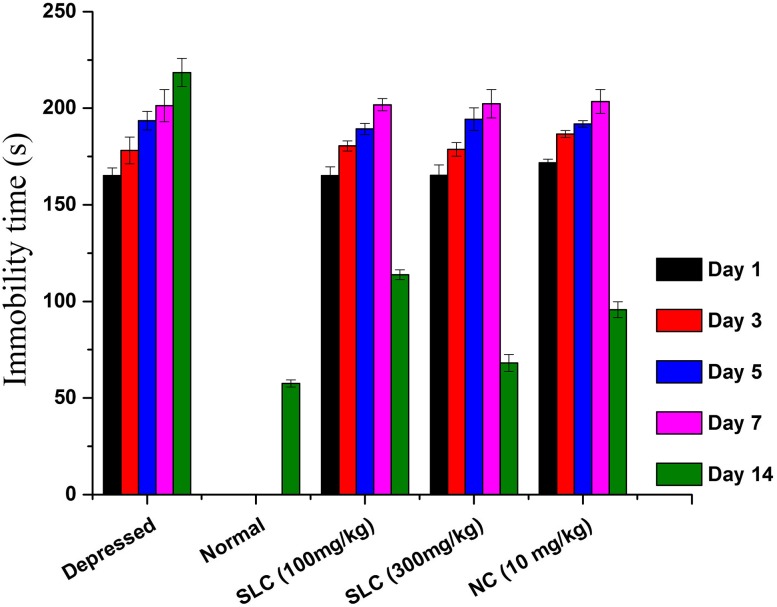
Effect of lithium carbonate in assaying the immobility time (FST) among depression-induced mice (*SLC* standard lithium carbonate, *NC* nanocomposite)

**Fig. 7:**
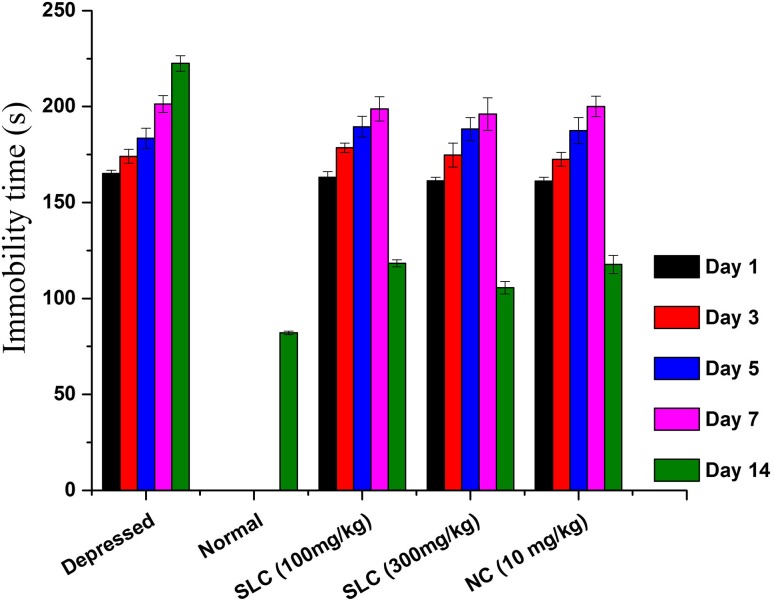
Effect of lithium carbonate in assaying the immobility time (TST) among depression-induced mice (*SLC* standard lithium carbonate, *NC* nanocomposite)

The forced swim test (FST) and tail suspension test (TST) are the most widely used tests of antidepressant action and are also used to infer “depression-like” behavior. Immobility in forced swim and tail suspension test showed significant impairment before treatment in both the standard and nanocomposite groups on 7th day as compared to 1st day. The post-treatment with standard drugs and their relative nanocomposites shortened the immobility period in the animal despair studies performed and exhibited a dose-dependent antidepressant activity. The results of immobility time are presented in Figs. 6 and 7. The mean value of immobility of animals treated with the standard lithium carbonate and nanocomposite simultaneously were found to be significant in comparison to control (*p* < 0.05). The standard lithium carbonate with a dose of 10 mg/kg body weight was found to be effective with a least immobility period and exhibited a minor difference to the normal mice. The treated nanocomposite relatively reduced the duration of immobility and established a distinct level in the dosage of 10 mg kg/body weight of the animals, which maintained them active. The nanocomposites exhibited positive results that managed the immobility period of animals. The degree of immobility was in accordance to the study of Prabhjot et al. (2014) who, experimented on Tramadol HCl (centrally acting synthetic opioid) as NP-loaded in situ gel against depression.

### Histopathological assay

**Fig. 8:**
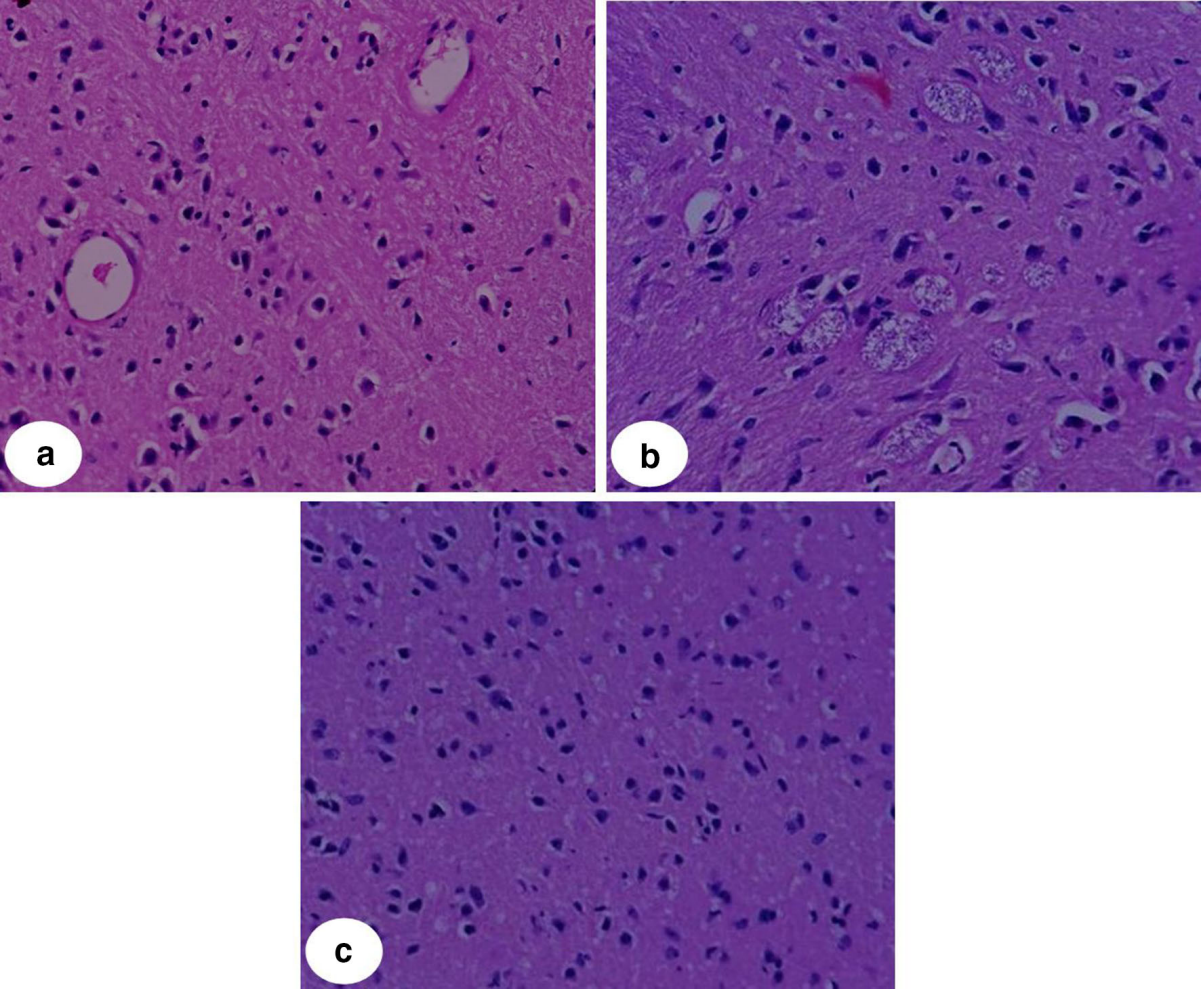
Histopathological changes in cerebral cortex regions of the **a** treated brain with nanocomposites with less-prominent gliosis and degenerative change **b** depression-induced mice exhibiting gliosis and degenerative patterns, **c** normal mice (x400)

The histopathological study provides a certainty in the focal degeneration of cerebral cells which is due to the oxygen depletion in the brain cells. Chronic reductions in cerebral blood flow and brain energy metabolism result in progressive dysfunction of neurons, leading to degenerative changes in glial cells. The constraint in blood flow further has caused microvascular constriction and glucose and oxygen delivery (Tsuchiya et al. 1993; Kerins et al. 2000). These results in a pathological condition are known as gliosis which is illustrated in the histology of the depressed animals. Incessant behavioral activity induces depression in a greater grade which is reflected in the histology of cerebral cortex. Forebrain sections in the cerebral cortex of the depressed mice showed (Fig. 8) an increase in degenerative changes and gliosis. Oxidative damage induced to brain implicates reduction in brain energy metabolism which causes more microvascular constriction and reduction in blood flow that leads to microgliosis. The nanocomposites alleviate and restore the persuaded gliosis that occurs in cerebral cells. Figure 8c shows less and mild changes in the cerebral cortex that illustrates the significance of lithium carbonate-loaded nanocomposites. The histopathological study confirms the ability to reverse the degenerative changes, gliosis that can reinstate psychological illness.

## Conclusion

In conclusion, the work furnishes the characterization of the prepared nanocomposites that assays the morphology and drug–polymer interaction which further promotes to evaluate its drug parameters. Chitosan nanoparticles had exhibited a definite ability to associate with lithium carbonate with a final concentration of 3.0 mg/mL that has the proficiency to reduce the dosage level thereby restraining adverse effects. The prepared nanocomposite with the biopolymer chitosan enhances drug delivery and serves as a carrier for which, the polymer–drug interaction is an essential factor. The release profile of lithium carbonate from nanoparticles facilitates a sustained continuous release which can improve oral absorption. The highly positive zeta potential of nanoparticles reveals better stability hoisting as a prominent drug. The in vivo studies show a significant alteration in the biochemical parameters of oxidative enzymes that has been attested by the histopathological studies. The nanocomposite administered in the depression-induced animal models has reversed degenerative changes and gliosis. This study furnishes a prelude to elevate polymeric nanocomposites as efficient brain drug to restore mental illness.
